# Bounded Responsibility and Bounded Ethics of Science and Technology

**DOI:** 10.1007/s10516-020-09514-7

**Published:** 2020-09-16

**Authors:** Günter Abel

**Affiliations:** Institute of Philosophy, Technical University Berlin (TUB), Sekr. H 72; Room H 7150/51, Straße des 17. Juni 135, 10623 Berlin, Germany

**Keywords:** Normativity in ethics of science, Bounded responsibility, Bounded ethics, Principle of reflective equilibrium, Internal and external responsibilities, Uncertainties in the sciences, Climate research

## Abstract

The leading question of this paper is: Where does the normativity of the ethics of science and technology come from? This is a challenging question given that the traditional reservoirs of convenience (like metaphysical universalism) are no longer at our disposal the way they used to be. The paper is divided into eight sections: (1) It is specified what challenges a non-foundationalist justification and normativity has to meet. (2) A three-dimensional conception of responsibility is developed based on the human triangular I–We–World relations. (3) The concepts of bounded responsibility and bounded ethics of science and technology are formulated. (4) The principle of reflective equilibrium is introduced as a principle of rationality, and it is shown how this principle generates rational and reasonable justifications in the ethics of science and technology. (5) Against this background, a reconception of internal and external responsibilities of science is given. (6) The type of responsibility demanded is exemplified by today’s climate research. (7) The paper argues for a hand-in-hand model of uncertainties in the sciences and for ethical obligations to preserve the conditions of human life on earth. The ethical argument is spelled out in terms of ethical care, preservation, and precaution. (8) Additionally, some arguments are developed to answer the question of why it is reasonable at all to preserve human life on earth.

## What is at Stake?

In what follows, what will be at stake is responsibility in science and technology. More precisely, what is at stake in these fields are, on the one hand, specific questions regarding e.g. the different types of responsibility in science and technology, the basic rules of proper scientific practice, or the validity of scientific standards. On the other hand, there is the more fundamental question of *where* the normativity of the ethics of science and technology comes *from* and how it functions. This *Where*-*from*-question can be completed by the two further questions of *why* we need an ethics of science and technology at all and *what* exactly it is *for*. These three *W*-questions are basal. They reach beyond the specific questions mentioned above.

The challenge that the answers to these latter questions face can be emphasized as follows: Where does normativity come from when the classical and metaphysical reservoirs of convenience are no longer at our disposal the way they used to be? Such reservoirs traditionally used to be available, e.g., in religious instances or in a realm of previously secured ethical values and norms, in other words: in a metaphysical universalism. How do we free ourselves from the predicament of requiring normativity but not being able to import it from some external domain or external authorities?

But fortunately there are starting points that can help us out here. In this paper, I will particularly elucidate the following three starting points with focus on the ethics of science and technology.

First (a) we develop the conception of a human-oriented *bounded responsibility* and *bounded ethics* focused on domain-specific as well as concrete problem solving (Sects. 2 and 3). Then (b) we can, from a reflective point of view, go back into what I call *practice*-*internal normativity* (Sects. 3 and 4). With this term I want to address that type of normativity that should not be conceived in the sense of *external* rules and criteria, but in the sense of human and practice-*internal* rule following. That is the kind of normativity that we are interested in under the sketched conditions of the loss of a metaphysical comfort zone. The thesis is that this normativity is always already presupposed in precisely those human life, knowledge, science, and technology *practices* in which it is internally embodied and relied upon. The normativity we are interested in here is always *internal*, not external normativity.

Finally (c) we are, as finite human beings, systematically (not only contingently) cut off from a realm of *universalistic* norms and values. But at the same time we can shape and justify the relation between, e.g., our considered moral judgments and our general ethical principles in such a way that the validity as well as the justification of the values and norms is warranted. This is where the *Principle of Reflective Equilibrium* comes into play. I would like to apply this principle in view of the above-mentioned predicament, i.e. the loss of metaphysical comfort zones, as well as in view of the foundation of *bounded responsibility* and *bounded ethics* (Sect. 4).

Trivially, in this altered perspective everything depends on the realization that it is ourselves, as humans, who actively, in an evidence-based and problem-solving way, and in virtue of our practice-internal normativities try to maintain our human life practices and expand our competencies and our scope of action.

Likewise, we can name further areas of the image and concept of man illustrated in this paper. Furthering these areas is, humanely viewed, a central task of the ethics of science and technology. These areas include the possibilities of: (a) taking an active part in shaping and orienting our life practices; (b) furthering milieus in which our human capacities can develop; (c) opening spaces for the generation of something new, i.e. milieus of creativity; (d) warranting human education in the age of scientific and technological civilization; (e) opening, securing, expanding, and strengthening the realms of human autonomy; and (f) keeping open and strengthening our human disposition to make rational and reasonable decisions. This latter point must be emphasized particularly in view of irrational and unreasonable decisions, which are always possible. Reasonability is a human disposition, not a natural property. We can choose rationality and reasonability or not. The humane ethics of science and technology must not lose sight of these crucial points.

But let us first focus on the concepts of *three*-*dimensional* responsibility (Sect. 2), *bounded* responsibility, and *bounded* ethics (Sect. 3). In all following deliberations (Sects. 2 to 8), I will also make reference to the three major challenges of our time: climate change, pollution, and pandemics. We will be able to elucidate the conception of the responsibility of science and technology using the example of climate research (Sect. 6). These elucidations will be carried out in such a way that we, on the one hand, not only do not deny uncertainties in the sciences and its modelings, but rather make them more precise and spell them out explicitly (Sect. 7). On the other hand, however, it is emphasized that the ethical obligation to preserve the conditions of human life by means of appropriate measures of preservation, precaution, and care is independent from the remaining uncertainties in scientific theories, models, and chaotic developments of dynamic systems. Some deliberations on the questions of why it is rational and reasonable at all to preserve the conditions of human life on this planet constitute the conclusion of this paper (Sect. 8). Throughout these points and sections the above-mentioned *normativity* plays the ultimately decisive role in the ethics of science and technology.

## Three-dimensional Responsibility

In what follows, I want to make the case for a three-dimensional scenario that constitutes a significant expansion of the classical field of ethical responsibility, care, preservation, and precaution. In this view, the transition from an ethics particularly focused on subjectivity and intersubjectivity into the *triangular* responsibility and ethics of the *I*–*We*–*World/Nature relations*, i.e. into a, in this sense, three-dimensional responsibility and three-dimensional ethics, is imperative.

Responsibility is a complex and multilayered phenomenon. In sum, the word means to stand up for one’s own actions, ascribe them to oneself or other persons or institutions, and account for them. And thus it means to be held accountable for the consequences of one’s own actions (or one’s omission to act). This aspect of standing up for one’s actions is completed by the aspect of taking responsibility in the sense of actively participating in the development of solutions to concrete problems. The latter aspect stands out especially when we have to face the challenges of life, society, and nature with future-oriented solutions. Facing responsibility and contributing to solutions to those challenges is what is at stake. The second sense of the talk of responsibility is currently important in view of the challenges in terms of climate change, environmental pollution, and pandemics. Responsibility in the first sense means that science and technology must be accountable for their actions and artifacts. In the second sense of the talk of responsibility we expect science and technology to provide future-oriented and sustainable solutions (e.g., to develop and provide a vaccine in the case of the current coronavirus pandemic).

As human beings we do not live secondarily, but primordially in our above-mentioned triangular human I–We–World/Nature relations. In this sense, man is the relational being par excellence. Obviously, the single components of these triangulations cannot be strictly isolated against one another. I deliberately chose the triangular model. We know the triangle as a musical instrument. One of this instrument‘s characteristic features is that when one of its three sides is struck, the other two sides chime as well. Transferring this triangular model, we can assume that the inseparability of our human I–We–World/Nature relations also has consequences for our moral and ethical responsibilities in the world, towards other persons, ourselves, and nature. If one of the relations becomes topical or even conflictive, the other two are always already at play.

The triangular model transports another important message. We are always, even in view of ever trans-subjective ethics, dealing with not only a bipolar boundedness of I–We relations, but with a tripolar boundedness in the sense of our human I–We–World/Nature relations. This tripolar understanding of the talk of trans-subjective is important in this context because the bipolar talk of an inter-subjective ethics (e.g., an inter-subjective discourse ethics) primarily focuses on the relations between the subjects involved and thus, so to speak, runs the risk of leaving out nature. In the proposed three-dimensional model of the I–We–World/Nature relations, however, nature is included from the start (which is particularly relevant these days in view of the current environmental, climate-, and pandemic-related challenges). We do not have to bring nature into play in a second step (or by means of an additional material argument). Nature is always already included, whether we like it or not, and it has ethical values of its own.

In the light of this expansion, I would like to argue the case for this kind of *three*-*dimensional* conception of responsibility and ethics. This point is crucial not only in view of the current debates on climate change, environmental issues, and pandemics. It is also relevant particularly because from now on the question of the normativity of the ethics of science and technology does not only refer to each one of us as an individual first-person subject. And neither does it simply refer to our fellow human beings as co-subjects. Instead, nature, surroundings, and environment as well as human physical health are included as legitimate recipients of responsibility and ethics. They are conceived as new and equal residents in the house of ethics. If these findings seem trivial,—then all the better.

## Bounded Responsibility and Bounded Ethics

The human triangular I–We–World/Nature relations can also be examined in terms of their interconnections, entanglements, situatedness, embodiments, and entrenchments, i.e. in terms of their different types of boundedness. This internal boundedness to concrete contexts, challenges, situations, and problems requires, when it comes to responsibility and ethics, what we can call *bounded responsibility* and *bounded ethics*.^1^ These two conceptions are not defined by or dependent on abstract, formal, and universalistic principles. Rather they can be seen as conceptions deeply rooted in our human life practices themselves.

This fundamental change of perspective also bears consequences for the answers to our three W-questions regarding the normativity of the ethics of science and technology. The vector of attention is shifting. The consequence is that we should not expect or look for the solutions to problems of our life, knowledge, and science practices in a realm of a universalistic responsibility, ethics, and quasi-eschatological theories. We should rather, being the finite thinking subjects we are, focus our attention on the concrete problems and challenges right in front of our eyes that require sustainable solutions (e.g. environmental or climate- and pandemic-related problems). Whether or not the concepts of bounded responsibility and bounded ethics can provide ethically responsible, justified, and justifiable solutions to the respective domain-specific and concrete problems is thus of crucial importance. This is the first sense of the talk of bounded responsibility and bounded ethics.

A second sense of this talk lies in the fact that the concrete, considered, and ethically responsible solutions to problems must have the status of justified or, if necessary, justifiable solutions. Consequently, justified and considered judgments and actions are of particular importance in our life practices. Given that scenario, we have to ask whether there is a principle which could take up the functions of the former metaphysical comfort zone and science-related ethical universalism. I would like to propose “*The Principle of Reflective Equilibrium*” as an appropriate candidate for these functions (see upcoming Sect. 4).

A third sense of the talk of ‘bounded responsibility’ and ‘bounded ethics’ results from the irreducible but heterogeneous variety of concrete and highly diverse challenges in the context of ethical responsibilities. Thus it is important to know what kind and domain of concrete challenges in terms of responsibility and ethics we are dealing with, for example: (a) with responsibility in the domain of human health (e.g. a doctor’s decision for or against performing a high-risk surgical procedure); (b) with responsibility for a section of nature and environment (e.g. with questions concerning the use of chemicals in farming and agriculture); (c) with a specific responsibility in view of animal welfare (animal ethics); or (d) with the effects a certain type of diet can have on our health and wellbeing. Simply put, the profiles, problems, challenges, and solutions of moral and ethical responsibility can present themselves very differently in the various fields, respects, and perspectives depending on whether we are dealing with bounded responsibility in cases of, e.g., medical, ecological, animal-related, dietary, or other problems and challenges.

Likewise, the responsibilities of, e.g., a pilot, a teacher, a climatologist, a football coach, a conductor, a prosecutor, or a member of another profession are obviously very different. Hoping for a universalistic authority, i.e. the one and only ultimate authority, or a ‘tertium comparationis’ in these cases would not only be misleading. It would be ultimately irresponsible and could not be reasonably justified.

We certainly can and must ask and determine what characteristics the different types of profession-bounded responsibility have in common. But our experiential realities and real life practices require (first of all and basically) multi-dimensional, concrete, and complex responsibilities of a cooperative and problem-solving kind, not one-dimensional, abstract, and universalistic ones. Critically viewed, it simply cannot be made plausible to conceive of the irreducibly many different types of responsibility merely as special cases of the one and only universalism, without typical individualities and differences among one another, and to try to subsume them under such a construction like ‘the one universalistic concept of responsibility’. The alternatively proposed idea of bounded responsibility and bounded ethics thus also aims at bringing the individuality of challenges concerning ethics and responsibility as well as the individuality of ethos- and ethics-bounded problem solving into the focus of attention. Bounded responsibility and bounded ethics are, so to speak, about nothing less than staying true and committed to humankind and the preservation of the conditions of human life.

It must be noted here that the sketched sense of domain-specific, problem-related, and bounded ethics is not simply to be understood in the sense of the talk of ‘applied ethics’. Even the term ‘applied ethics’ is slightly misleading. For this wording presupposes the distinction between ‘pure and universalistic ethics’ on the one hand and an ‘application of this ethics to concrete situations and challenges’ on the other hand. Precisely this dualism, however, is what the concept of bounded responsibility and ethics is *not* about. Rather, the talk of the boundedness and justification of, e.g., considered moral judgments, ethical principles, actions, and concrete solutions has to do with those considered (and thus not random and arbitrary) judgments, decisions, and actions required in the concrete human practices, situations, problems, and challenges themselves (as, e.g., in the intensive care unit of a hospital). Hence, we could also speak of ‘*situated responsibility and ethics*’.

## The Principle of Reflective Equilibrium

As soon as we need justification in a situation where questions or even conflicts in the sketched I–We–World/Nature triangulation occur, we search for a method of justification and rational conflict resolution. Against the background of the described scenario (Sect. 3), this needs to be a method and principle that can perform the task without recourse to an abstract universalism and without universalistic and ultimate justifications. I believe that the most promising principle for this task is the Principle of Reflective Equilibrium.^2^ This principle seeks (by means of reciprocally adjusting and adapting the respective components in question) to either prove a given state of balance, of equilibrium (and thus of coherence) of the entire system to be already justified, or to create new justifications and secure them for the time being.

The method of reflective equilibrium can successfully be applied in the following domains: (1) in the domain of inductive logic (as illustrated by Nelson Goodman in view of the relation between considered judgments and deductions on the one hand, and general rules on the other); (2) in the domain of the sciences (regarding the relation of single scientific judgments to the general rules of scientific theories, which Goodman has also illustrated); (3) in the domain of moral philosophy and ethics (regarding the relation of everyday moral judgments on the one hand and general moral and ethical rules and principles on the other hand).

I explicitly want to include in this list (4) the points of overlap between ethics and science and thus the domain of the ethics of science and technology. Accordingly, I would like to use the principle of reflective equilibrium in view of the question of which normative requirements the ethics of science must meet in order to contribute to the orientation of humans and their actions. Furthermore (5), I believe that we can, with recourse to the principle of reflective equilibrium, provide a satisfying characterization of the triangular dynamics and states of our human I–We–World/Nature relations. The principle can in my opinion also (6) successfully be applied in view of the processes and states of successful and/or unsuccessful and conflicting human communication and cooperation. The two latter domains (5) and (6) primarily have to do with the important role of the principle of reflective equilibrium in the field of language-, sign-, and action-based equilibria of comprehension and action.^3^

The relation between a generally accepted everyday moral judgment on the one hand and an (also accepted) general ethical principle on the other hand can help illustrate what the principle of reflective equilibrium is about. Think of, e.g., the relation between the moral judgment ‘The fact that Peter pays for the oranges he put in his basket at the grocery store makes it a morally good action’ and the general ethical principle ‘Things that are for sale have to be paid for’. As long as the relation between both sides functions directly, fluently, connectively, and (for the time being) unquestionably, there is no problem. But questions, irritations, disturbances, or conflicts regarding the individual judgment or the general principle and/or their relation can always arise. Think of, e.g., the relation between the moral judgment ‘Isolating the elderly in order to protect them from contracting the coronavirus is a morally good action’ (which judgment has actually been made in the context of the corona pandemic) and the general ethical principle ‘Humans must not be confined’. In this case, the relation between the moral judgment and the general ethical principle does not function fluidly anymore. As a consequence, we no longer view the judgment as justified, but rather exclude it from the category of considered and justified judgments.

Such conflicts not only occur in the relations between moral judgments and ethical principles. As mentioned earlier, they can also be found within the sciences. Think of, for instance, the relation between physical judgments of the type “The astronomical observations XYZ and the calculations ABC show that the earth revolves around the sun” and the (pre-Copernican) principle “The earth is the static center of the universe that is orbited by the sun”. In this case of conflict between considered judgment and general principle (and in contrast to the above-mentioned conflict between moral judgment and general principle), it is a well-known historical and systematic fact that not the object-related propositional scientific sentence, but the (up to that point justified) general theory of the universe lost its validity.

In cases where questions, disturbances, and conflicts in the relation between moral judgment and ethical principle arise (or between a physical sentence and a scientific theory), we search for a method to either restore or rearrange the fluid functioning of the relations (for the time being). This is the crucial achievement of the principle of reflective equilibrium. In the reflective process characteristic of this principle, the considered judgments/sentences are pondered against each other and, if possible, brought into a coherent balance, a reflective equilibrium, by means of adjustments. Both the general principle’s and the considered judgment’s being justified, in ethics as well as in science, derive from the prevailing circumstances, i.e. from those life or knowledge practices under which they are established, justified, and accepted. I would like to call this the *pragmatic dimension* of the principle of reflective equilibrium. Nelson Goodman illustrated the crucial mechanism of the dynamic and adjusting processes of this relation and thus was the first one to formulate the principle of reflective equilibrium: “A rule is amended if it yields an inference we are unwilling to accept; an inference is rejected if it violates a rule we are unwilling to amend. The process of justification is the delicate one of making mutual adjustments between rules and accepted inferences; and in the agreement achieved lies the only justification needed for either.”^4^

At this point I would like to emphasize the internal relation between the principle of reflective equilibrium and the talk of bounded responsibility and bounded ethics (introduced in Sect. 3). As finite thinking subjects—God does not need a principle of reflective equilibrium!—, we are committed to the humane principle of equilibrium as well as to the humane boundedness, situatedness, and entanglement in the circumstances of our life practices. In this perspective, the principle of equilibrium embodies two different performance profiles at the same time: on the one hand (a) the principle includes the request to make explicit a so far assumed state of being justified; on the other hand (b) the principle helps, if necessary, to generate (or reject) new justifications in the face of new challenges. The first case has to do with being able to make explicit, when requested, the state we are usually in (in the above-mentioned example: trusting the considered judgment just as much as the general principle). That also means having to show that both (in our example: judgment and principle/rule) are valid and well-justified in their relation to each other and thus in an equilibrium we accept.

Such a state deserves to be called a (for the time being) satisfying and justified state. “For the time being” in this context means as long as no critical requests arise and the directness, fluency, connectiveness, and self-evidence of the assumption of being justified are warranted. But as soon as questions, disturbances, irritations, and conflicts occur, we apply the principle of reflective equilibrium. If in the course of the then made adjustments, balances, and improvements no equilibrium is achieved, we are usually willing to modify and revise both judgment and rule and sometimes even abandon them and replace them with different and new judgments and rules. We find ourselves in a quasi-revolutionary situation when not just one of the two sides (sentence or theory; judgment or principle), but the entire construction itself cannot be maintained anymore. In this case, new paradigms and new background assumptions are required.

In both respects (making explicit a given being justified; generating required justifications) the principle of reflective equilibrium can be conceived as a human *principle of rationality* (see Abel 2016). The principle is also important in view of our appropriate reactions to entirely new and challenging situations as well as in view of the opening of a new space of possible and creative solutions. I would like to bring *creativity* into play here in the sense that the processes of reciprocal adjustments (conceived as processes of improvements of balance regarding the change and interplay of the components involved) can lead to creative, new, and innovative improvements.

Such improvements can, e.g., also be required in the sciences (and thus in, e.g., the climate, environmental, and pandemic sciences). In the sciences, an improvement usually consists in an increase in the precision of the used models, theories, and simulations. Such improvements can be imperative in the face of new data or new empirical observations. The new data and observations are from then on included as components in the (nowadays primarily mathematical and statistical) models. The activities involved in these processes can be conceived as modeling-relevant creativities.^5^

But the principle of reflective equilibrium can not just be conceived of as a *principle of rationality*. It can, at the same time, be understood as a *principle of humanity*. This is possible simply because the principle is a crucial human (and not a divine) method. And the processes of reciprocal adjusting as well as the states of successful balance are characteristic of and desirable to us humans across the entire spectrum of our triangular I–We–World/Nature relations and thus across the entire spectrum of our human experience, perception, speech, thought, action, and creativity. Accordingly, the idea of an ethics of science and technology is essentially a human-related, human-bounded, human-based, human-oriented, and human-orienting matter. In other words: it is a humane matter. Humanity, rationality, and normativity go hand in hand. An ethics of science and technology must be ultimately grounded and anchored in human life, i.e. in our human life practices and circumstances, if such an ethics is supposed to have normative power for us. And it must, at the same time, refer to those life, knowledge, science, and technology practices if it indeed wants to be relevant and orienting for the human triangulation of the I–We–World/Nature relations (and not just get lost in intellectual finger exercises).

My answer to the three *W*-questions posed in Sect. 1 regarding the normativity of the ethics of science and technology (*Where* does it come *from*?, *Why* normativity at all?, *What* is normativity *for*?) is thus the following: The required normativity and ethical responsibility can, critically viewed, only be searched for and only be found where we are dealing with what I earlier called the *practice*-*internal normativity and responsibility* of our human life, communication, cooperation, action, and orientation *practices*. The required normativities and responsibilities are not to be found in a separate and external realm of metaphysical and universalistic ideas, but internally in our triangularly bounded life practices themselves. We can reflect ourselves into the individual rules, logics, and mechanisms of these practices. And we do so with the intention of finding and elucidating the human origins and goals of the normativities and responsibilities and making them a guideline for the ethics of science and technology. The application of the principle of reflective equilibrium makes it possible and also necessary to examine whether or not the background assumptions brought to light by such a reflection are justified and whether or not they can be justified. The principle of reflective equilibrium thus explicitly includes the principle of critique. Critically viewed, however, this critique and examination itself can always and only be internal, not external critique.^6^

## The Internal and External Responsibilities of Science

Within the described scenario (Sects. 1 to 4), we can now address other and more specific aspects in terms of responsibility in science and technology. These include the distinction between two types of responsibility, (a) the science- and technology-*internal* responsibility and (b) the science- and technology-*external* responsibility.^7^ This distinction also constitutes the crucial difference between the *ethos* of individual scientists on the one hand, and the *ethics* of the sciences as specific scientific disciplines on the other hand.

Science-internal/ethos responsibility means the obligation towards the rules of proper scientific work with the goal of generating, expanding, and securing knowledge as opposed to merely subjective opinions and ideologies. Internal/ethos responsibility in the sense of, e.g., scientific best practice, a code of norms, and an ethics of professions inside the scientific community includes, among others, the following values and norms: the obligation to justify beliefs, hypotheses, theories, and models; the obligation not to manipulate data; including new data and observations in the examination; ensuring the transparency of data collection and data evaluation; revising and modifying previous models and theories in the face of new data and observations; the obligation to subject one’s own theories and models to discussion and criticism; the willingness to consider alternative models and theories; the obligation to reexamine previously used models in the light of new insights, data, and observations; making improvements in terms of an increase in the precision of models and theories; the willingness to extend, modify, revise, and, if necessary, abandon a hypothesis, theory, model, or simulation.

In terms of science-external/ethical responsibility of scientists and the sciences, different types of responsibility can be distinguished as well. The following four types help us meet some of the challenges of modern societies.

(1) A first type of responsibility can be found in the area of *actively intervening research* (e.g. in scientific and technological experiments, in which human beings are the immediate test subjects and thus the direct objects of research, like in the coronavirus vaccination tests). (2) A second type of responsibility can be found in those areas of scientific research that deal with *consequences* in the areas of, e.g., biotechnology, gene technology, nuclear technology, or nanomedicine. (3) A third type refers to responsibility in terms of consequences of certain *man*-*made scientific and technological developments* themselves. Think of, e.g., the consequences of scientific or technological developments for climate change and environmental pollution.

(4) Finally, as already mentioned, a fourth type of responsibility science and technology have to face must be emphasized. Science and technology have an obligation to make substantial and sustainable contributions to (a) the preservation, precaution, and improvement of the *conditions of human life* on earth and to (b) the shaping of the *future of modern societies*.

It must be emphasized, however, that the remarks regarding the fourth type of responsibility by no means imply a desire to maneuver science and technology into the role of political decision makers. That is definitely not the case. The social and political decisions regarding concrete measures and programs lie in politics, not in science. Science and technology must, however, face the responsibility of providing contributions and recommendations in view of rational, intelligent, and reasonable decisions in the political realm. And they do so in various ways and by providing various scientific and technological competencies, disciplines, practices, and professions. The handling of questions concerning climate change and the coronavirus pandemic are current examples of this.^8^ Making a recommendation in no way means entering the field of political consulting. Rather, a recommendation from the sciences or technologies is, so to speak, the spelling out of an answer to the (inner-scientific and inner-technological) question of what the respective scientific or technological analyses, results, and predictions mean and what consequences follow from them. It would be unscientific and ethically irresponsible if the sciences tried to keep their findings contained to themselves (e.g. in the case of climate change or pandemic research).

Earlier we made a distinction between internal and external responsibilities of the sciences. But the four illustrated types of responsibility show that, across the board, internal and external responsibilities are at the same time tightly connected and interwoven. It should also have become obvious that the distinction of these two ways of responsibility does not suggest that science and technology can be viewed as *ethically neutral affairs*. Science and technology must take a perspectival responsibility for their own practices, processes, and products and justify them if requested. Neutrality can, strictly speaking, neither be directly achieved here nor is it the crucial strategic goal and norm. Rather, the *epistemic*, the *epistemological,* and the *ethical goal and norm* is constituted of the preservation, protection, and improvement of the human life practices and the conditions of human life on earth.

On more detailed examination of this task, we must distinguish a variety of different types, tasks, and conflict situations of science-*internal* as well as of science-*external* responsibilities and their overlaps. This variety cannot be presented and discussed in detail here. Hans Lenk has addressed this subject and made some fine-grained conceptual distinctions in this context (see Lenk, e.g., 1991, p. 61 ff. and p. 64 and recently 2020). Using the question of responsibility in climatology as an example, I would like to point out just one aspect. As Lenk correctly emphasizes, we are not only dealing with direct responsibilities, but also with “indirect” responsibilities. Indirect responsibilities are not immediately given by the action situation itself. Examples of “science-induced long distance effects” can be used to illustrate what is meant by indirect responsibilities. Think of the delayed and remote effects of interventions in nature, e.g. of the use of ecologically harmful pesticides or plastic materials.

But the sciences view themselves as obligated to the spirit of enlightenment and thus essentially embody three key obligations that must be explicitly emphasized. First (1), there is the obligation to provide the best scientific results possible and to maintain their priority over mere opinions and politically motivated ideologies. Second (2), there is an obligation to provide the best possible rationality in terms of intellectually convincing and empirically rich arguments. And the third (3) obligation, as I would like to emphasize once more, is to contribute to the concrete improvement of life and to the solving of problems, crises, and unforeseen situations in the age of science and technology by using the scientific and technological tools and resources available at the time.

The legitimacy, normativity, and benefit of science and technology are based on these three pillars. And the obligation of scientists to actively take part in public debates (e.g. currently in debates on climate change, environmental issues, and the coronavirus pandemic) is based on these pillars, too. The sciences must, according to their own self-conception, always be involved in order to not just be a vacuous ceremony of hypotheses, models, theories, and simulations, but beneficial to our human life, knowledge, science, and technology practices.^9^

## Responsibility Exemplified by Climate Research

In the current (and often heated) discussions on climate research and its prognoses, the reference to uncertainties concerning scientific explanations, theories, modelings, and forecasts often plays an important role. The situation is analogous in the case of pandemic-related debates. Uncertainties in the sciences are particularly brought up when, as in the case of so-called climate change sceptics, attempts to stabilize and positively influence climate development are rejected because of uncertainties regarding the theory. This is the case despite the fact that overwhelming majority of scientists not only recommend the respective analyses, diagnoses, therapies, and measures, but regard them as postulates that need to be realized as soon as possible. Otherwise, there could be devastating consequences for human life on earth and the planet itself. Regardless of these concerning findings, many climate change sceptics misinterpret the reference to uncertainties in the sciences as an excuse for inactivity or even as an ideological weapon.

It must be noted here that the *Intergovernmental Panel on Climate Change (IPCC)* explicitly discusses the question of how reliable, precise, and resilient the current models of climate science are.

But even if we cannot definitively eliminate uncertainties from science and technology, this does not mean, in terms of ethical responsibility, that we are not committed to the ethical principle of prevention, precaution, and care. This ethical obligation towards our own human conditions of life receives its legitimacy from a different source than the degree of reliability of statistical methods and scientific (mathematical) modelings. Legitimacy rather arises from the ethical commitment to prevention, precaution, and care particularly from two directions: firstly (1), from the answer to the question of what actions (and omissions of actions) are ethically imperative in order to preserve and secure the conditions of our functioning human life practices; and secondly (2), from the answer to the question of what is imperative under the rule of human rationality, of everyday practical prudence (*phrónesis*), and of reasonableness and what is not.

The rule of prudence must by no means be misunderstood as a capitulating retreat to ethically mediocre slogans such as “The ends justify the means” or “The justification of moral judgments and ethical principles is ultimately a matter of cost–benefit calculation”. We have heard the latter slogan particularly in connection with ethical utilitarianism and the min–max rule in the context of the rational choice theory. In contrast however, the rule of prudence does not formulate a mediocrity, but a human peak performance. In book VI of his *Nicomachean Ethics*, Aristotle emphasizes the practical prudence (*phrónesis*) in its own relevance towards and in addition to theoretical knowledge (episteme). Practical prudence means the ability to appropriately act in concrete individual situations with regard to the factors, goals, and insights internally related to our human conduct of life and thus to our life practices in the sense of a pragmatic knowledge. Practical prudence is, in this sense, always *bounded prudence*. And the question of the legitimacy of this type of obligation to provide care, precaution, and prevention (given, e.g., the results of today’s climate research) is an everyday relevant practical and thus ethical question. Moreover, this ethical question is primarily *not* a question of the sciences and hence cannot be answered with scientific means, methods, and models.

I would like to illustrate this point using the example of the obligations of a physician. Kant used this example in his *Critique of Pure Reason* (Kant 1956, B 852f). A slightly modified version of this example can help illustrate the point. A patient suffering from a severe headache consults a doctor. The doctor’s initial diagnosis is migraines, but he is not entirely sure. He tries to comfort (or even reject) the patient by telling him that there still are too many uncertainties regarding his diagnosis. And it could very well be the case that another doctor might make a more positive diagnosis. Thus he, the doctor, is still too far away from an absolutely certain and perfect knowledge required in advance in order to confirm the diagnosis and treat the headache appropriately. And thus the doctor, in his own opinion, does justifiably (sic!) not believe that it is his duty to actively care and take precautions in the given case. He feels sorry for the patient, but his obligation to provide absolute certainty regarding his knowledge forces him to inaction, in ethical terms, to the omission of a treatment. In view of this example we have to explicitly emphasize that the doctor obviously is, due to his medical ethos and due to the ethics of care, precaution, and prevention, obligated to act. What do we learn from this example?

First of all, it is needless to say that the physician from our example would likely be prosecuted for failure to provide assistance. This way, the entire process turns from a process of ethics into a legal matter. For as a doctor, he *must* act. He may not act from a position of absolute certainty (since such certainty is never actually available anyway). Rather, his actions are based on what we can, with reference to Kant, call the “pragmatic belief” (Kant 1956, B 852). This term describes the pragmatic interaction of sufficiently subjective and sufficiently justified belief in order to enter into the action (even if the doctor does not have absolute knowledge in this case). Neither the doctor nor the patient need any further justification.

## Uncertainties in the Sciences

Uncertainties and susceptibility to error in the sciences (and thus in climate, environmental, and pandemic science, too) cannot be entirely eliminated. All science is subject to uncertainties. And there is also the fact that the individual sciences themselves are primarily defined by their limits and boundaries. But at the same time, all sciences constantly try to improve their hypotheses, models, and theories and try to avoid problems that can arise from limited perspectives. In what follows, we will therefore focus on illustrating the inner connection of (a) the quasi-natural uncertainties in the sciences and (b) the measures of prevention, precaution, and care that are ethically imperative in order to prevent damage to or even the destruction of the conditions and foundations of human life. With this question, we are obviously dealing with an important point of overlap of scientific and theoretical analysis and ethical obligation.

In terms of the sciences, we can, according to Dagfinn Føllesdal, distinguish at least three types of uncertainties: (a) uncertainties in theories, (b) uncertainties in models, and (c) uncertainties in chaotic systems.^10^

On (a): *Uncertainty of theories*:—In scientific theories (e.g. theories on climate change), hypotheses are made and then tested in terms of whether or not they match our empirical observations. Uncertainty exists here in the sense that it is always possible that the prognoses made by means of mathematical models and statistical methods do not match our empirical observations.

Also, competing (and sometimes even mutually exclusive) theories, in the sense of Willard Van Orman Quine’s thesis of underdeterminacy, always remain possible. According to this thesis (which I strongly agree with), every theory is systematically underdetermined in terms of the data base it receives its input from (e.g. an astrophysical theory on the beginning and development of the entire universe). There is always a relation between a “meager output” and a “torrential input”. The question following from this finding is the question of “in what ways one’s theory of nature transcends any available evidence” (Quine 1969, p. 83). This transcending is always given and necessary, no matter how large an amount of data we are talking about. For, according to Quine, it is not an underdetermination that can ultimately be empirically corrected somehow. It is rather a logical underdeterminacy. And within this open realm of underdeterminacy, two different (and maybe even mutually exclusive) but equally valid theories can be formulated.

It must be noted, however, that this finding does by no means lead to a complete and terminal uncertainty in the sciences. Rather, it is crucial that we still can and must be able to compare and distinguish between good and less good hypotheses, models, and methods. The crucial criterion for preference cannot be the recourse to absolute and perfect knowledge. The criterion is rather provided by the answer to the question of whether or not a hypothesis, model, or theory is able to explain our observations more precisely and coherently. If a theory succeeds to do so, we are justified in our belief that it is a good theory (for the time being). The ethos- as well as ethics-based obligation in terms of responsibility resulting from this scenario of uncertainties is the obligation to constantly test the hypotheses, models, and theories with new empirical observations and new data and thus modify and improve them.

On (b): *Uncertainty of models*:—A model is, as Føllesdal correctly emphasizes, a representation of something that highlights certain characteristics and disregards others. Today, particularly mathematical and statistical models are predominant in the sciences. They operate with various parameters and items that are considered relevant and incorporated in the equations of the model as well as in the modeling. In many cases, however, these are not items we can empirically observe, but estimated items that we adopt from other relevant fields and then incorporate and apply in our models.

Obviously even the smallest alterations of these items can have great effects on the course and results of mathematical and statistical modeling. In the current epidemiological modeling of the course of the coronavirus pandemic, e.g., this is the case depending on whether the reproduction factor R (i.e. the number indicating how many new infections are statistically caused by a single infected person) is increased or decreased. If the parameter R increases, e.g. from R = 1 to R = 3, and this new figure is incorporated in the equations of the model, this can result in significant alterations in terms of the previously predicted number of new infections. The related curve of the predicted course of the pandemic would rise exponentially instead of linearly. Against this background it becomes clear that models and modelings are always accompanied by uncertainties. **“**If there are enough equations and appropriate parameters, a model can be matched with almost any data” (Føllesdal, p. 3).

The calculated predictions made by means of the model are supposed to match our most recent data and observations. In this sense, scientific modelings are always dynamic. As Føllesdal (p. 4) says, “we are more likely to trust a model that matches observations that were not known yet at the time the model (or theory) was formulated”. This certainly also holds true for today’s models of climate change and pandemic development.

We could even go one step further. Because no matter how tight a modeling’s restrictions in terms of the relation between model and reality may be, there always remains the possibility of various equally legitimate relations between model and reality. And thus there are always (as Hilary Putnam has shown) various equally valid satisfaction relations and satisfaction objects.^11^ This degree of uncertainty internally connected to models themselves cannot be eliminated, neither empirically (no matter how much data we possess) nor theoretically (no matter how strict the formal restrictions of our models may be).

On (c): *Uncertainty due to the chaotic development of systems*:—Føllesdal (p. 6 f) reminds us of the famous butterfly scenario formulated by the meteorologist Edward Lorenz (1917–2008). As is well-known, Lorenz simply wanted to re-run a weather simulation on his computer. But Lorenz did not start this second run at the beginning of the simulation, but at a later stage at which he entered the numbers his printer had printed out for this stage. To Lorenz’s surprise, the second run lead to results that were significantly different from the ones of the initial simulation. And the reason for this was as surprising as it was simple: Lorenz’s computer had calculated to six decimal places while his printer had only printed out the first three decimal places. Thus, a number like 0,506127 was abbreviated and used in the calculations as 0,506. And this relatively small difference between the two numbers lead to an entirely different course and meteorological result of the simulation. This way Lorenz had demonstrated that even the smallest alteration of the initial conditions can have great consequences and that even an optimal model cannot accurately predict the weather for more than a short period of time.

In his famous paper, Lorenz writes:

If the flap of a butterfly’s wings can be instrumental in generating a tornado, it can equally be instrumental in preventing a tornado. (…) Since we do not know exactly how many butterflies there are, nor where they are all located, let alone which ones are flapping their wings at any instant, we cannot, if the answer to our question is affirmative, accurately predict the occurrence of tornados at a sufficiently distant future time. (Lorenz 1972, p. 1f)

This butterfly effect is an example of a phenomenon that occurs in non-linear and dynamic systems.

We know uncertainties of the described type as well as the associated measures of care and precaution from our everyday life. Føllesdal (p. 12 f) uses the example of getting fire insurance to illustrate this point. The risk of my house burning down is relatively small, but if it actually happened, it could possibly be devastating for me. That is why it is obvious to me that I get appropriate insurance and believe that my decision is rational and reasonable. In situations like this, in which what is at stake is the prevention of real possible future catastrophes or even the destruction of the conditions of human life, the illustrated principle of ethical care, prevention, and precaution, and thus the responsibility of care, prevention, and precaution, come into play (Lenk 1991, p. 64).

Føllesdal emphasizes an aspect that is crucial in this context. When it comes to the possibility of harm, there must be a realistic possibility, not just a merely theoretical possibility. He uses a nice example to illustrate this. It may be compatible with scientific theories and theoretically possible that I will be hit by a large meteorite in the near future. But it would not be rational, prudent, or reasonable to get an insurance for this case, since the probability of such a collision actually happening is extremely small. That would be just as unreasonable as the even further-reaching decision to live my entire life under the assumption that I have to avoid such a collision at any cost.

## Epilogue: Why is it Reasonable at all to Preserve the Conditions of Human Life on Earth?

Imagine a doubting Thomas comes along and poses the question of why it is reasonable and rational to preserve the conditions of human life on earth at all. The answer to this question seems instantaneous and obvious. Who honestly does not know why we should protect and preserve ourselves, other persons, our environment, as well as planet earth?! As soon as we have to explicitly spell out this self-evidence, however, it seems that we (in Wittgenstein’s words) do not know the answer anymore. In what follows, I would like to illustrate some aspects that concern our self-interest as human beings and can be understood, directly or indirectly, as an affirmative answer to this provocative question. It is presupposed in this answer that this affirmation of life and not self-destruction is a primordial characteristic and presupposition of human life itself. This life-internal presupposition needs no proof. Rather, it is always already internally included in what we call living our lives. Demanding any additional proof would be what Kant rightfully called a “scandal of philosophy and human reason in general” (Kant 1956, B XXXIX).

1. If we do not take measures of care and precaution in terms of, e.g., climate change (and accordingly in terms of viral pandemics), the threat of harm becomes a real possibility that not only affects individual and isolated areas of the conditions of human life on earth. It rather affects the conditions of life of the human species itself as a whole and thus ultimately the survival of humanity as a whole. Putting that on the line would not exactly be a sign of reasonableness now, would it?
2. Humankind survives in the course of nature’s survival. This finding is trivial. At the same time, we must recall another triviality, i.e. the fact that humankind needs nature, but nature does not need humankind. Do we really want to abandon our human self-manifestation on earth in light of these findings? By the way, in this context antique Stoicism introduced the concept of “oikeiosis”, i.e. the survival instinct. In the Stoa, this concept describes the reasonable attitude and wish to bring one’s own life and one’s own self-preservation as well as one’s self-care into a well-proportioned balance with nature. As is well known, this important principle of the Stoa can be understood as an invitation to live one’s life “in appropriate harmony with nature (*homologoumenos te physei zen*)”. It is safe to say that this principle is as relevant today as ever.
3. There is a lot of evidence that suggests that the active shaping of our relations to ourselves, others, and the world is characteristic of human life. What is at stake in these activities is certainly not just the mere existence and continuation of biological, organic human life. It is rather the internally connected pursuit of *a good life* (which idea was established by Aristotle). The punchline of this idea of a successful and good life can pointedly be formulated as follows: from the moment on I (as a biological, organic individual) wish to preserve my own biological, organic existence, I am always already tied to the idea of living a good life.
  One could say that we orient ourselves towards well-proportioned relations as well as successful states of equilibrium within the illustrated triangulation of our human I–We–World/Nature relations. The idea of a good life is bounded to its embodiment in this triangulation. *Good life* in this sense is always *bounded good life*. It is a triangulation-based, triangulation-bounded, and triangulation-oriented life. And a good life always already includes other people, the world, nature, the environment, and the entire planet earth. Why then should we unnecessarily put this reality of life at stake or even abandon it entirely?!
4. Kant formulated his famous categorical imperative for human beings as follows: Act in such a way that the maxims of your actions were to become a general law. On the grounds of the sketched triangulation of the human I–We–World/Nature relations, this imperative always also includes, next to the responsibility for oneself, the responsibility towards other persons and nature. This imperative is directly relevant with regard to the internal as well as the external responsibility of science and technology. With special focus on today’s scientifically and technologically determined realities of life, the following version of the categorical imperative can be formulated: ‘Act in such a way that the maxims of your actions were to become a general law that takes into account the preservation and expansion of human life practices and conditions of life and provides possibilities for living a good life on earth.’^12^

## Footnotes

1 I use these two expressions with reference to Herbert A. Simon’s concept of “bounded rationality”. See Simon (1956). The concept of bounded rationality was significantly extended and expanded in Gigerenzer and Selten (2001).

2 This principle was formulated by Nelson Goodman and John Rawls and then further developed by Catherine Z. Elgin. See Goodman (1983) and Rawls (1971). Rawls (1971, p. 20) explicitly makes reference to Goodman’s method in order to emphasize that making reciprocal adjustments between general principles and considered judgments is not only characteristic of moral and legal philosophy, but fundamental to the entire field of deductive and inductive rules and conclusions. On the epistemological aspect of this principle, see Elgin (1996, chap. IV) and (2003).

3 On this last type of equilibrium, particularly the equilibrium of comprehension and the equilibrium of action, see Abel (2018a).

4 Goodman (1983, p. 64). John Rawls, who coined the term “reflective equilibrium”, explains his choice as follows: “It is an equilibrium because at last our principles and judgments coincide”. And it deserves to be called “reflective since we know to what principles our judgments conform and the premises of their derivation.” See Rawls (1971, p. 20).

5 On the role of creativity in this context, see Abel (2009).

6 On the concept of *practice*-*internal normativity*, see Abel (2018b). This paper examines the question of the practice-internal normativity particularly in view of (a) the fluid functioning of speaking a language and (b) successful knowing-how (e.g. riding a bicycle or tying a necktie). In the present paper, I expand this concept of practice-internal normativity to our human life practices in total.

7 On this distinction see Lenk (1991, p. 56 ff).

8 On the latter see, e.g., the recommendations of the Leopoldina (German National Academy of Sciences; April 13th, 2020) under the title *Coronavirus-Pandemie—Die Krise nachhaltig überwinden (Coronavirus Pandemic—Sustainably Overcoming the Crisis)*. See on this also the statement of researchers of the Helmholtz Association under the title *Systemische Epidemiologische Analyse der COVID-19-Epidemie (Systemic Epidemiological Analysis of the COVID-19 epidemic*, also April 13th, 2020).

9 On the role and task of philosophy in the public, social, and political domain, see Abel (2020/forthcoming).

10 See Føllesdal (2011). This paper has only been published in Norwegian language so far. I would like to thank Rune Nyrup for translating it to German for my personal use. I would also like to thank Dagfinn Føllesdal for proofreading and authorizing Nyrup’s translation. In what follows, I will quote this German version of Føllesdal’s paper. The quotation page numbers refer to this so far unpublished German version by Nyrup. I would very much welcome a published English and German version of this important paper.

11 See Putnam (1983). See also Abel (2008), in which I illustrate the relation of model and reality as a *sign-based* and *interpretation-determined relation*. On this, see also the index entry “Modell” (“model”) in Abel (2018c).

12 Hans Jonas (1984, p. 11) has proposed an imperative of responsibility for the age of technology. It reads as follows: “Act so that the effects of your actions are compatible with the permanence of genuine human life.” I agree with this formulation as it is. Jonas, however, bases this imperative on a number of presuppositions that require further discussion. I explicitly disagree with Jonas’ ontological justification of our norms and values as well as with his general strategy of basing ethics in metaphysics. But this subject cannot be discussed here.
